# Association of Primary Care Continuity With Home Dialysis, Transplantation, and Utilization of Medical Services for Patients Starting Hemodialysis

**DOI:** 10.1016/j.xkme.2025.101015

**Published:** 2025-04-19

**Authors:** Cole S. Wyman, Maya Djerboua, Kristin K. Clemens, Ziv Harel, Manish M. Sood, Samuel A. Silver

**Affiliations:** 1 Queen’s University, Kingston, Ontario, Canada; 2 ICES, Ontario, Canada; 3 Division of Endocrinology and Metabolism and Department of Epidemiology and Biostatistics, Western University, London, Ontario, Canada; 4 Division of Nephrology and Li Ka Shing Knowledge Institute, St. Michael’s Hospital, University of Toronto, Toronto, Ontario, Canada; 5 Department of Medicine and Clinical Epidemiology Program of the Ottawa Hospital Research Institute, University of Ottawa, Ottawa, Ontario, Canada; 6 Division of Nephrology, Kingston Health Sciences Center, Queen’s University, Kingston, Ontario, Canada

**Keywords:** Hemodialysis, end-stage kidney disease, primary care

## Abstract

**Rationale & Objective:**

Primary care may help patients starting dialysis with emotional support and access to health care services. It is unknown whether consistently visiting the same primary care physician (PCP) can strengthen patient confidence to select home dialysis, help facilitate medical appointments for transplantation, or increase care access.

**Study Design:**

A population-based retrospective cohort study.

**Setting & Participants:**

Patients initiating maintenance hemodialysis from 2007 to 2017 in Ontario, Canada.

**Exposure:**

High PCP continuity using the usual provider of care index (an established measure of PCP continuity), defined as >75% of PCP visits with the same PCP in the 2 years before dialysis initiation.

**Outcomes:**

Primary outcomes were time to home dialysis (peritoneal or hemodialysis) and transplantation. Secondary outcomes included specialist visits, cancer screening, influenza vaccination, and measures of diabetes care.

**Analytical Approach:**

Propensity scores to match patients with high and low PCP continuity.

**Results:**

We identified 9,530 matched pairs. High PCP continuity was not associated with increased home dialysis (14.0 events per 100 person-years vs 14.0 events per 100 person-years; subdistribution hazard ratio 1.00; 95% CI, 0.97-1.04) or transplantation (4.3 events per 100 person-years vs 4.5 events per 100 person-years; subdistribution hazard ratio 0.97; 95% CI, 0.90-1.04). High PCP continuity was associated with greater colon cancer screening (hazard ratio 1.07; 95% CI, 1.01-1.14), influenza vaccination (hazard ratio 1.33; 95% CI, 1.27-1.39), and comprehensive diabetes care (hazard ratio 1.23; 95% CI, 1.14-1.33).

**Limitations:**

Residual confounding is possible.

**Conclusions:**

High PCP continuity before dialysis initiation was not associated with increased utilization of home dialysis or transplantation but was associated with greater colon cancer screening, influenza vaccination, and comprehensive diabetes care. Additional work is needed to clarify how primary care may best benefit this patient population.

## Plain Language Summary

Patients transitioning to maintenance hemodialysis require comprehensive health care. We investigated how closer relationships with a primary care physician (PCP) may complement nephrologists in caring for this patient population. We used administrative databases in Ontario, Canada, to conduct a retrospective population-based study, assessing how continuity with one PCP influenced the uptake of home dialysis, kidney transplantation, and patient access to health care services. We found that high PCP continuity before dialysis initiation was not associated with increased transition to home dialysis or kidney transplantation but did lead to increases in colon cancer screening, influenza vaccination, and diabetes assessment. Further work is required to determine how PCPs can help nephrologists best serve the multifaceted needs of patients transitioning to maintenance hemodialysis.

The transition to maintenance hemodialysis is a challenging time for patients, who may benefit from the increased support and care coordination provided by a close relationship with a primary care physician (PCP) in addition to nephrologist care for their hemodialysis needs. We recently demonstrated that high PCP continuity, defined using the usual provider of care index (>75% of PCP visits with the same PCP in the 2 years before dialysis initiation) was not associated with a lower risk of mortality or all-cause hospitalizations.^1^ This lack of benefit may have been the result of isolated primary care management having limited ability to improve these outcomes, especially in a population with a high burden of comorbid conditions.

Primary care management has been shown to increase utilization of health care services for patients on maintenance dialysis, such as screening for depression, cancer screening, and influenza immunization.^2^^,^^3^ Therefore, we hypothesize that PCP involvement may help patients treated with dialysis with emotional support and access to health care services. Support from consistently visiting the same PCP may strengthen patient confidence and self-management, increasing selection of home dialysis.^4^^,^^5^ A closer PCP-patient relationship may also help arrange the specialist visits, medical tests, and psychological support required to complete the steps required for kidney transplantation.^6^

In this population-based study from Ontario, Canada, we sought to further explore how a close PCP relationship could complement nephrologist care and potentially benefit patients on maintenance hemodialysis. Our objective was to determine whether high PCP continuity of care before dialysis initiation is associated with increased access to home dialysis or kidney transplantation. We also measured access to specialist care, cancer screening, influenza immunization, and diabetes care to provide an overview of how consistent visits with the same PCP affect medical service utilization for this vulnerable patient population.

## Methods

### Study Design

We conducted a population-based propensity score matched cohort study within Ontario, Canada (population >14 million). We used administrative data from ICES databases linked using unique encoded identifiers.^7^ The ICES is an independent, nonprofit research institute with legal status under Ontario’s health information privacy law that allows it to collect and analyze health care and demographic data without consent for health system evaluation and improvement. The use of data in this project was authorized under section 45 of Ontario’s Personal Health Information Protection Act, which does not require review by a research ethics board. The reporting of this study follows the reporting of studies conducted using observational routinely collected health data guidelines for observational studies (Table S1).^8^

### Databases

This study used several provincial-linked databases to define study criteria, exposures, outcomes, and covariates (Table S2). The Canadian Organ Replacement Register is a national database containing information about kidney replacement therapy and organ donation.^9^ The Ontario Health Insurance Plan database contains all Ontario physician claims, enabling the tracking of services including the location of the service (ie, outpatient or inpatient), date of the service, diagnosis, and procedure performed. The ICES physician database identifies physician characteristics and specialty (collected through periodic telephone surveys combined with billing information). We collected information about hospitalizations using the Canadian Institute for Health Information Discharge Abstract Database. We determined patient demographics and vital status through the registered persons database and area-level demographics from the postal code conversion files.

### Study Population

We identified patients aged ≥18 and ≤105 years in Canadian Organ Replacement Register who initiated maintenance hemodialysis from January 1, 2007, to December 31, 2017. The first hemodialysis session was the index date, determined through validated Ontario Health Insurance Plan physician billing codes and Canadian Institute for Health Information Discharge Abstract Database Canadian Classification of Health Interventions codes.^10^^,^^11^

We excluded individuals if they had missing or invalid data including ICES’ unique identifier (given to individuals with a valid Ontario Health care number), age, or sex, died before or on the index date, were non-Ontario residents, or received a kidney transplant in the 5 years before the index date. We also excluded patients with <3 PCP visits in the 2 years before the index date (because at least 3 visits are needed to ascertain PCP continuity) and patients residing in long-term care (due to differences in primary care provision in that setting).

### Exposure

We classified high PCP continuity as a usual provider of care (UPC) index >0.75 (>75% of PCP visits were with the same PCP in the 2 years before dialysis initiation). We classified low PCP continuity as a UPC index ≤0.75. The UPC index is the most established tool for determining PCP continuity of care, and it is associated with high-quality physician-patient interactions.^12^^,^^13^

### Outcomes

The primary outcomes were time to home dialysis transfer (peritoneal or hemodialysis) and kidney transplantation ascertained from Canadian Organ Replacement Register. We analyzed these outcomes individually and adjusted for the competing risk of death. Secondary outcomes included specialist visits (cardiology, endocrinology, psychiatry, and palliative care), cancer screening (breast, cervical, prostate, and colon),^14^ influenza immunization,^15^ and diabetes care (comprehensive assessments and vision tests).^16^ We ascertained specialist visits from Ontario Health Insurance Plan and ICES physician databases using a previously published algorithm to measure visits for palliative care.^17^ The remaining secondary outcomes were ascertained through Ontario Health Insurance Plan, and we also used the Ontario breast cancer program database to identify the presence of breast cancer screening (Table S2). Follow-up commenced at dialysis initiation and continued until December 31, 2019. Emigration from the province is the only reason for lost follow-up, and the rate is low (<0.5% per year).^18^

### Propensity Score Development

We developed a multivariable logistic regression model to estimate propensity scores for high PCP continuity.^19^ We selected covariates based on their clinical significance, which included age, sex, ethnicity, rural residence (community with population ≤10,000), socioeconomic status (by mean neighborhood income quintile), primary cause of kidney failure, comorbid conditions in the preceding 5 years, and hospitalizations and outpatient physician visits in the preceding 1 year. We also included PCP characteristics, such as age, sex, years since graduation, international medical school graduate, hospital affiliation, practice location (rural versus urban), and dialysis patient volume. We used a structured, iterative approach to refine this model and obtain balance within the matched pairs.^20^ We measured covariate balance by the standardized difference, with an absolute standardized difference of >10% representing meaningful imbalance.^21^ We used a greedy matching algorithm without replacement using the logit of the propensity score (±0.2 standard deviation [SD]) to match patients with high PCP continuity 1:1 to patients with low PCP continuity. We forced an exact match on sex and diabetes to facilitate reporting of the cancer and diabetes secondary outcomes.

### Statistical Analyses

We summarized baseline characteristics by UPC status using descriptive statistics, expressing continuous variables as mean ± SD or median (25th and 75th percentile) and categorical variables as frequency (percentage). For the primary outcomes, we derived subdistribution hazard ratios and 95% confidence intervals using Fine-Gray proportional hazards models with a robust sandwich variance to account for correlation within the matched pairs.^22^ For the secondary outcomes, we used the Andersen-Gill model to allow for multiple events per patient, censoring for death.^23^ We also descriptively reported secondary outcomes for a subset of patients still alive on maintenance hemodialysis at 5 years in case the frequency of secondary outcomes was lower because of premature death and to ascertain medical service utilization in healthier patients. We considered a 2-sided *P* < 0.05 as statistically significant and performed all analyses using SAS version 9.4 (SAS Institute).

## Results

### Cohort Build and Patient Characteristics

**Figure 1:**
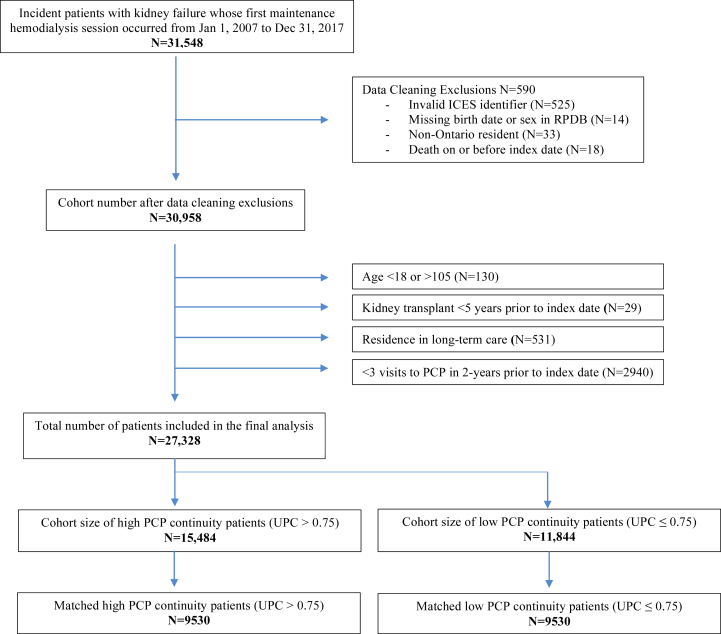
Cohort build, PCP, primary care physician; UPC, usual provider of care index.

From January 1, 2007, to December 31, 2017, a total of 31,548 patients with kidney failure initiated maintenance hemodialysis in Ontario, Canada (Fig 1). The most common reason for exclusion was <3 visits to any PCP in the 2 years before dialysis initiation (n = 2,940, 9%). Of the 27,328 patients included in the study cohort, 15,484 (57%) had a UPC index >75% and 11,844 (43%) had a UPC index ≤75%.

In the unmatched cohort of 27,328 patients (Table 1), patients with high PCP continuity had lower Charlson comorbidity scores (3.6 ± 1.8 vs 4.1 ± 2.0) and fewer comorbid conditions (chronic liver disease, chronic obstructive pulmonary disease, congestive heart failure, dementia, and depression). High PCP continuity patients also had fewer encounters with the health care system, including fewer PCP visits (11.0 ± 10.4) versus (13.6 ± 14.8), specialist visits (internal medicine, nephrology, and cardiology), and hospitalization days over the previous year (5.9 ± 13.9) versus (11.5 ± 21.9), and less home care involvement.

**Table 1:** Characteristics of Patients Initiating Maintenance Hemodialysis in Ontario, Canada, Before Propensity Score Matching

Baseline Characteristics	Low Continuity (n = 11,844)	High Continuity (n = 15,484)	Standardized Difference
Demographics			
Age (y), median (IQR)	66 (54-76)	69 (59-78)	0.21
Female, n (%)	4,647 (39.2)	5,965 (38.5)	0.02
Ethnicity, n (%)			
White	8,151 (68.8)	11,007 (71.1)	0.05
Indian subcontinent	860 (7.3)	960 (6.2)	0.04
Black	694 (5.9)	763 (4.9)	0.04
Asian	539 (4.6)	1,105 (7.1)	0.11
Other/unknown	1,600 (13.5)	1,649 (10.7)	0.09
Income quintile, n (%)			
1 (lowest income)	3,223 (27.2)	3,788 (24.5)	0.06
2	2,600 (22.0)	3,545 (22.9)	0.02
3	2,258 (19.1)	2,968 (19.2)	0.003
4	1916 (16.2)	2,815 (18.2)	0.05
5 (highest income)	1,785 (15.1)	2,309 (14.9)	0.004
Rural residence, n (%)	1,470 (12.4)	1,792 (11.6)	0.03
Rostered to a primary care physician, n (%)	9,597 (81.0)	13,383 (86.4)	0.15
Primary cause of kidney failure, n (%)			
Diabetes	4,341 (36.7)	5,777 (37.3)	0.01
Kidney vascular disease	1,646 (13.9)	2,708 (17.5)	0.10
Glomerulonephritis/autoimmune	1,271 (10.7)	1,602 (10.4)	0.01
Cystic kidney disease	351 (3.0)	519 (3.4)	0.02
Other	2,619 (22.1)	2,873 (18.6)	0.09
Unknown	1,616 (13.6)	2005 (13.0)	0.02
Year of cohort entry, n (%)			
2006	6 (0.1)	14 (0.1)	0.02
2007	669 (5.7)	1,373 (8.9)	0.12
2008	640 (5.4)	1,302 (8.4)	0.12
2009	686 (5.8)	1,344 (8.7)	0.11
2010	788 (6.7)	1,378 (8.9)	0.08
2011	899 (7.6)	1,304 (8.4)	0.03
2012	1,011 (8.5)	1,290 (8.3)	0.01
2013	1,194 (10.1)	1,451 (9.4)	0.02
2014	1,388 (11.7)	1,493 (9.6)	0.07
2015	1,441 (12.2)	1,534 (9.9)	0.07
2016	1,598 (13.5)	1,477 (9.5)	0.12
2017	1,524 (12.9)	1,524 (9.8)	0.10
Comorbid conditions, n (%)			
Cancer	4,480 (37.8)	5,862 (37.9)	0.001
Chronic liver disease	1,516 (12.8)	1,479 (9.6)	0.10
Chronic obstructive pulmonary disease	1,490 (12.6)	1,449 (9.4)	0.10
Chronic pain	426 (3.6)	347 (2.2)	0.08
Congestive heart failure	4,513 (38.1)	5,045 (32.6)	0.12
Dementia	729 (6.2)	604 (3.9)	0.10
Depression	596 (5.0)	383 (2.5)	0.14
Diabetes	7,110 (60.0)	9,216 (59.5)	0.01
Hypertension	10,569 (89.2)	14,238 (92.0)	0.09
Myocardial infarction	1,353 (11.4)	1,472 (9.5)	0.06
Peripheral vascular disease	836 (7.1)	1,079 (7.0)	0.004
Stroke/transient ischemic attack	901 (7.6)	927 (6.0)	0.01
Modified Charlson comorbidity index, mean ± SD	4.1 ± 2.0	3.6 ± 1.8	0.22
Health care utilization in previous year, median (IQR)			
No. of visits to all primary care physicians	9 (5-17)	8 (5-14)	0.12
No. of visits to most frequent primary care physician	5 (2-9)	7 (4-13)	0.51
No. of internal medicine visits	2 (0-7)	1 (0-5)	0.23
No. of nephrology visits	6 (3-10)	6 (3-9)	0.10
No. of endocrinology visits	0 (0-1)	0 (0-0)	0.07
No. of cardiology visits	2 (1-6)	2 (1-5)	0.15
No. of geriatric medicine visits	0 (0-0)	0 (0-0)	0.10
No. of psychiatry visits	0 (0-0)	0 (0-0)	0.12
No. of hospitalization days over previous year	3 (0-14)	0 (0-6)	0.36
Home care, n (%)	3,975 (33.6)	4,028 (26.0)	0.17
Physician characteristics			
Age (y), mean ± SD	52.3 ± 11.8	55.3 ± 10.3	0.28
Male, n (%)	8,325 (70.3)	12,055 (77.9)	0.17
Number of years since graduation, mean ± SD	25.9 ± 12.5	29.3 ± 10.7	0.30
International medical graduate, n (%)	2,993 (25.3)	3,438 (22.2)	0.07
Hospital affiliation, n (%)	4,931 (41.6)	5,089 (32.9)	0.18
Rural practice, n (%)	1,155 (9.8)	1,464 (9.5)	0.01
Dialysis patient volume in previous year, n (%)			
0	2,027 (17.1)	2,649 (17.1)	0.00
1	2,306 (19.5)	3,366 (21.7)	0.06
2	1,947 (16.4)	2,991 (19.3)	0.08
3	1,500 (12.7)	2,149 (13.9)	0.04
4	1,008 (8.5)	1,397 (9.0)	0.02
5	3,056 (25.8)	2,932 (18.9)	0.17

*Note:* Health care utilization in previous year with 0 median visits, summarized as categorical variables, n (%). No. of endocrinology visits: low continuity 0 = 8,801 (74.3), 1 = 921 (7.8), 2 = 719 (6.1), and ≥3 = 1,403 (11.9); high continuity 0 = 11,894 (76.8); 1 = 1,194 (7.7); 2 = 962 (6.2), and ≥3 = 1,434 (9.3). No. of geriatric medicine visits: low continuity 0 = 10,793 (91.1), 1 = 418 (3.5), 2 = 193 (1.6), and ≥3 = 440 (3.7); high continuity 0 = 14,495 (93.6), 1 = 452 (2.9), 2 = 176 (1.1), ≥3 = 361 (2.3). No. of psychiatry visits: low continuity 0 = 10,915 (92.2), 1 = 314 (2.7), 2 = 162 (1.4), and ≥3 = 453 (3.8); high continuity 0 = 14,715 (95.0), 1 = 286 (1.9), 2 = 134 (0.9), and ≥3 = 349 (2.3).

We matched 9,530 patients with high PCP continuity 1:1 to similar patients with low PCP continuity (Table S3). After propensity score matching, the number of PCP visits over the previous year were (11.7 ± 11.5) in the high PCP continuity group and (11.7 ± 11.8) in the low PCP continuity group. Most of these visits in the high PCP continuity group were with the same PCP (10.5 ± 10.4) compared with the low PCP continuity group (6.1 ± 6.5). Most PCPs in both the high and low continuity groups had ≤2 patients treated with dialysis in their practice (10,672/19,060, 56%), though 22% in each group had dialysis patient volumes 5. The total person-years of follow-up were 30,723 years in the high PCP continuity group (median 2.8, IQR 1.3-4.6 years) and 30,608 years in the low PCP continuity group (median 2.8, IQR 1.2-4.6 years).

During the follow-up period, the high PCP continuity group continued to have more continuity of care with their PCP with an average of 40.4 ± 53.1 total PCP visits, of which 16.4 ± 26.4 were with the UPC physician. Corresponding visits in the low PCP continuity group included an average of 40.4 ± SD 52.3 total PCP visits, of which 8.8 ± SD 18.0 were with the UPC physician.

### Outcomes

Relative to low PCP continuity of care (Table 2), high PCP continuity was not associated with increased home dialysis transfer (14.0 events per 100 person-years versus 14.0 events per 100 person-years; subdistribution hazard ratio 1.00; 95% CI, 0.97-1.04) or kidney transplantation (4.3 events per 100 person-years versus 4.5 events per 100 person-years; subdistribution hazard ratio 0.97; 95% CI, 0.90-1.04).

**Table 2:** Association of Primary Care Physician Continuity With Home Dialysis and Kidney Transplantation in Propensity-Matched Patients (N = 19,060)

Outcome and Exposure	No. (%) of Events	Events per 100 Patient-Years	Subdistribution Hazard Ratio (95% CI)
Home dialysis			
Low continuity	2,930 (30.8)	14.0	Referent
High continuity	2,939 (30.8)	14.0	1.00 (0.97-1.04)
Kidney transplantation			
Low continuity	1,384 (14.5)	4.5	Referent
High continuity	1,329 (13.9)	4.3	0.97 (0.90-1.04)

There was no association between high PCP continuity of care and specialist visits (Table 3), including cardiology (hazard ratio 1.01; 95% CI, 0.98-1.05), endocrinology (hazard ratio 1.01; 95% CI, 0.93-1.09), psychiatry (hazard ratio 0.84; 95% CI, 0.68-1.02), and palliative care (hazard ratio 1.02; 95% CI, 0.87-1.19). There was no difference in cancer screening, except high PCP continuity was associated with increased colon cancer screening relative to low PCP continuity (0.11 events per person-year versus 0.10 events per person-year; hazard ratio 1.07; 95% CI, 1.01-1.14). For diabetes management, high PCP continuity was associated with increased comprehensive diabetes care assessments (0.55 events per person-year versus 0.45 events per person-year; hazard ratio 1.23; 95% CI, 1.14-1.33) but not vision screening (1.40 events per person-year versus 1.42 events per person-year; hazard ratio 0.98; 95% CI, 0.92-1.05). The largest effect size for any medical service was influenza vaccination, for which high PCP continuity was associated with increased immunization relative to low PCP continuity (0.29 events per person-year versus 0.22 events per person-year; hazard ratio 1.33; 95% CI, 1.27-1.39).

**Table 3:** Association of Primary Care Physician Continuity With Specialist Visits, Cancer Screening, Influenza Immunization, and Diabetes Care in Propensity-Matched Patients (N = 19,060)

Outcome and Exposure	No. of Events per Patient-Year	Hazard Ratio (95% CI)
Specialist visits		
Nephrology		
Low continuity	54.09	Referent
High continuity	53.66	0.99 (0.98-1.01)
Cardiology		
Low continuity	4.33	Referent
High continuity	4.38	1.01 (0.98-1.05)
Endocrinology		
Low continuity	0.84	Referent
High continuity	0.85	1.01 (0.93-1.09)
Psychiatry		
Low continuity	0.51	Referent
High continuity	0.43	0.84 (0.68-1.02)
Palliative care		
Low continuity	2.94	Referent
High continuity	2.99	1.02 (0.87-1.19)
Cancer screening		
Mammography (women only)		
Low continuity	0.16	Referent
High continuity	0.17	1.07 (0.99-1.16)
Papanicolaou testing (women only)		
Low continuity	0.17	Referent
High continuity	0.17	1.03 (0.93-1.14)
Prostate-specific antigen testing (men only)		
Low continuity	0.08	Referent
High continuity	0.08	1.01 (0.86-1.18)
Colon cancer		
Low continuity	0.10	Referent
High continuity	0.11	1.07 (1.01-1.14)
Other preventative care		
Influenza immunization		
Low continuity	0.22	Referent
High continuity	0.29	1.33 (1.27-1.39)
Diabetes assessment (patients with diabetes only)		
Low continuity	0.45	Referent
High continuity	0.55	1.23 (1.14-1.33)
Diabetes vision screening (patients with diabetes only)		
Low continuity	1.42	Referent
High continuity	1.40	0.98 (0.92-1.05)

When describing medical service utilization for patients who survived for 5 years on maintenance hemodialysis (n = 4,014, Table S4), specialist visits were less, but cancer screening and preventative care was similar to the entire cohort (Table 3). Only a small proportion of patients who survived for 5 years on maintenance hemodialysis never saw a cardiologist (2% vs 7% in entire cohort). However, most patients did not visit with a psychiatrist (75% versus 82% in entire cohort). Cancer screening was also low, with 47% (68% in entire cohort) not receiving mammography, 60% (76% in entire cohort) not receiving Papanicolaou testing, 82% (89% in entire cohort) not receiving prostate-specific antigen testing, and 60% (76% in entire cohort) not receiving colon cancer screening. In patients with diabetes, 48% (64% in entire cohort) did not have a comprehensive diabetes care assessment, and 26% (49% in entire cohort) did not have a vision assessment. Many patients (46%) who survived 5 years of maintenance hemodialysis did not receive influenza immunization (vs 62% in entire cohort).

## Discussion

In our population-based cohort of over 19,000 patients who started maintenance hemodialysis from 2007 to 2017 in Ontario Canada, we found that high PCP continuity before dialysis initiation was not associated with subsequent home dialysis utilization or kidney transplantation. We did observed associations between high PCP continuity and increased colon cancer screening, influenza vaccination, and comprehensive diabetes care. These results should motivate further research and quality improvement initiatives to better define how primary care can best support patients on long-term maintenance hemodialysis.

Although we hypothesized based on previous literature that higher PCP continuity might increase emotional support and access to health care services,^2^, ^3^, ^4^ we observed few differences in medical service utilization between groups. It is possible that higher PCP continuity improved unmeasured determinants of health, such as self-management, patient confidence, and mental health, which are often barriers to selecting home dialysis or pursuing kidney transplantation^24^^,^^25^; however, these outcomes were also similar between the 2 groups. Given that over 75% of PCPs cared for 4 or fewer patients treated with hemodialysis, it is possible that PCPs are less familiar with the benefits and complications of home dialysis and kidney transplantation to offer patients the decision-making advice and support required. This may be an important care gap to explore further, given the increasing emphasis on kidney programs to grow home dialysis and kidney transplantation.^26^

Even though Ontario, Canada, has a single-payer, universal health care system and over 80% of patients in our study had access to a PCP, utilization of medical services was not much better than previously described experiences. According to United States Renal Data System reports,^27^^,^^28^ 47% of eligible patients undergo diabetes vision screening (vs 51% in our cohort), 31% of patients receive comprehensive diabetes care (versus 36% in our cohort), and 75% receive influenza vaccination (vs 38% in our cohort; lower likely due to some vaccinations occurring in hospitals and dedicated influenza clinics that are not captured by administrative data). In a Midwestern United States regional dialysis network,^3^ cancer screening based on United States Renal Data System coding for patients with primary care visits was 38% for breast cancer (vs 32% in our cohort), 18% for cervical cancer (vs 24% in our cohort), 13% for prostate cancer (vs 11% in our cohort), and 18% for colon cancer (vs 24% in our cohort). These comparable data suggest that simply having access to primary care, which is the case in Ontario, Canada, is insufficient to meaningfully increase health care utilization for patients treated with hemodialysis. Other potential reasons that have been proposed include patient- (eg, travel time and competing health demands), provider- (eg, nephrologists lack knowledge on primary care and PCPs lack knowledge on dialysis-related issues), and system-level barriers (eg, unclear role delineation between nephrologists and PCPs and absence of clinical practice guidelines).^29^, ^30^, ^31^

Smaller studies in the United States have demonstrated that patients treated with hemodialysis with primary care providers have statistically significant higher rates of influenza vaccination and screening for breast or colon cancer.^2^^,^^3^ Our data are consistent with these observations, though the effect sizes of high PCP continuity on breast and colon cancer screening are small. We found relative effect sizes for increases in influenza vaccination ranging from 27% to 39% and for increases in comprehensive diabetes assessments ranging from 14% to 33%. Both influenza vaccination and comprehensive diabetes care have been associated with reductions in morbidity for patients on hemodialysis,^32^, ^33^, ^34^ so these may be key areas to clarify and strengthen the role of primary care in the hemodialysis unit.

Another potential explanation for the modest effect of high PCP continuity on clinical outcomes and access to care is that there is no standard practice on how to provide primary care to patients treated with hemodialysis, including who should provide care (primary care provider vs nephrologist), where it should be provided (ambulatory clinic versus dialysis unit), and what it should consist of. One potential solution that has been piloted in the United States is creating a patient-centered medical home within the hemodialysis unit.^35^ This model incorporates a general internist (serving as a PCP), nurse coordinator, pharmacist, community health worker, and the hemodialysis team. In several ways, this care framework addresses the well-described barriers to primary care for patients treated with hemodialysis, including lack of patient time for PCP appointments, low PCP knowledge of dialysis-related issues, low nephrologist knowledge of primary care issues, lack of clarity in PCP versus nephrologist roles, and delayed information exchange between providers.^31^ Among 175 patients at 12 months postintervention, this primary care model improved several measures of health-related quality of life, including symptoms related to kidney disease.^36^ These promising results require replication, especially with the incorporation of PCPs into several end-stage kidney disease seamless care organizations in the United States,^37^ underscoring that primary care for patients treated with hemodialysis may have benefits beyond mortality, hospitalizations, and access to care. A major gap remains the lack of clear primary care guidelines for patients treated with hemodialysis, which are needed to increase uptake of potentially useful interventions (eg, home dialysis, transplantation, influenza vaccination, and diabetes care), decrease potentially unnecessary practices (eg, cancer screening in nontransplant candidates and duplicate tests), and incorporate symptom-based assessments (eg, depression screening and sexual health).

Strengths of this work are the inclusion of over 19,000 matched patients newly started on maintenance hemodialysis over a 10-year period. We focused on incident patients to reduce confounding and had very little missing data with virtually complete patient follow-up due to the nature of our administrative data holdings. Our datasets also allowed for the capture of sociodemographic characteristics, preexisting comorbid conditions, resource utilization, physician visits, and dialysis modality, which allowed for the adjustment of a broad array of potential confounders.

Our study also has limitations. First, there is no standard definition for PCP continuity of care, which did not include nurse practitioners or physician assistants. The UPC index, though, is one of the more established tools for determining PCP continuity of care and is associated with high-quality physician-patient interactions.^12^^,^^13^ Although the high PCP continuity group continued to have more UPC visits during the follow-up period relative to the low PCP continuity group, the number of visits with the same PCP in the high PCP continuity group declined over time, which could contribute to some of the lack of associations observed. Second, some residual confounding is likely despite our best efforts because administrative data limit the ability to capture the severity of comorbid conditions, which may affect PCP reasoning for providing medical services to certain patients versus others. Third, it is possible that high PCP continuity may improve quality of life measures that cannot be represented by measures of health care utilization or administrative data, which warrant further study. Finally, this study was conducted in a single Canadian province with a single-payer, universal health care system, where nephrologists usually provide some degree of primary care. Both groups also had regular visits with cardiology, endocrinology, and other specialists at baseline. Therefore, the results may not be generalizable to other populations, though rates of several health services were similar to those reported in the United States Renal Data System.^3^^,^^27^^,^^28^

Our population-based cohort of over 19,000 patients found that high PCP continuity for patients starting maintenance hemodialysis was not associated with increased utilization of home dialysis or transplantation, but was associated with greater colon cancer screening, influenza vaccination, and comprehensive diabetes care. We also observed that most PCPs only care for 1-2 patients treated with hemodialysis, which may make it challenging for education strategies alone to fill any knowledge gaps, including how to support patients considering home dialysis and kidney transplantation. These findings suggest that simply increasing primary care access or continuity for patients on maintenance hemodialysis may not necessarily confer better outcomes beyond what is already provided by nephrologists. Instead, additional work is needed to design primary care for patients treated with hemodialysis in a way that respects patient time, addresses PCP and nephrologist knowledge gaps, and fulfills patient primary care needs in an evidence-based and high-quality manner.
