# Supplementary material for: Association of Primary Care Continuity With Home Dialysis, Transplantation, and Utilization of Medical Services for Patients Starting Hemodialysis
Source: Kidney Med. 2025 Apr 19;7(6):101015. doi: 10.1016/j.xkme.2025.101015 (PMC12152618; doi:10.1016/j.xkme.2025.101015)
Supplement: Supplementary File (PDF) — Tables S1-S4. [file mmc1.pdf]

Table S1: Checklist of items from the REporting of studies Conducted using Observational Routinely-collected health Data (RECORD) guidelines for observational studies

	Item No.	STROBE items	Location in manuscript where items are reported	RECORD items	Location in manuscript where items are reported
**Title and abstract**					
	1	(a) Indicate the study's design with a commonly used term in the title or the abstract (b) Provide in the abstract an informative and balanced summary of what was done and what was found		RECORD 1.1: The type of data used should be specified in the title or abstract. When possible, the name of the databases used should be included.<br><br>RECORD 1.2: If applicable, the geographic region and timeframe within which the study took place should be reported in the title or abstract.<br><br>RECORD 1.3: If linkage between databases was conducted for the study, this should be clearly stated in the title or abstract.	Abstract<br><br>Abstract<br><br>Abstract

**Introduction**					
Background rationale	2	Explain the scientific background and rationale for the investigation being reported			Introduction
Objectives	3	State specific objectives, including any prespecified hypotheses			Introduction
**Methods**					
Study Design	4	Present key elements of study design early in the paper			Study design
Setting	5	Describe the setting, locations, and relevant dates, including periods of recruitment, exposure, follow-up, and data collection			Study design, population, exposure, outcomes
Participants	6	*(a) Cohort study* - Give the eligibility criteria, and the sources and methods of selection of participants. Describe methods of follow-up<br><br>*Case-control study* - Give the eligibility criteria, and the sources and methods of case ascertainment and control selection. Give the rationale for the choice of cases		RECORD 6.1: The methods of study population selection (such as codes or algorithms used to identify subjects) should be listed in detail. If this is not possible, an explanation should be provided.<br><br>RECORD 6.2: Any validation studies of the codes or algorithms used to select the population should be referenced. If	Supplement

		and controls<br><br>*Cross-sectional study* - Give the eligibility criteria, and the sources and methods of selection of participants<br><br>*(b) Cohort study* - For matched studies, give matching criteria and number of exposed and unexposed<br><br>*Case-control study* - For matched studies, give matching criteria and the number of controls per case		validation was conducted for this study and not published elsewhere, detailed methods and results should be provided.<br><br>RECORD 6.3: If the study involved linkage of databases, consider use of a flow diagram or other graphical display to demonstrate the data linkage process, including the number of individuals with linked data at each stage.	Databases and Study population<br><br>Figure 1
Variables	7	Clearly define all outcomes, exposures, predictors, potential confounders, and effect modifiers. Give diagnostic criteria, if applicable.		RECORD 7.1: A complete list of codes and algorithms used to classify exposures, outcomes, confounders, and effect modifiers should be provided. If these cannot be reported, an explanation should be provided.	Supplement
Data sources/ measurement	8	For each variable of interest, give sources of data and details of methods of assessment (measurement).<br><br>Describe comparability of assessment methods if there is			Databases, propensity score development, and supplement

		more than one group			
Bias	9	Describe any efforts to address potential sources of bias			Propensity score development and statistical analysis
Study size	10	Explain how the study size was arrived at			Figure 1
Quantitative variables	11	Explain how quantitative variables were handled in the analyses. If applicable, describe which groupings were chosen, and why			Propensity score development and statistical analysis
Statistical methods	12	(a) Describe all statistical methods, including those used to control for confounding<br>(b) Describe any methods used to examine subgroups and interactions<br>(c) Explain how missing data were addressed<br>(d) *Cohort study* - If applicable, explain how loss to follow-up was addressed<br>*Case-control study* - If applicable, explain how matching of cases and controls was addressed			Propensity score development and statistical analysis

		Cross-sectional study - If applicable, describe analytical methods taking account of sampling strategy<br><br>(e) Describe any sensitivity analyses			
Data access and cleaning methods		..		RECORD 12.1: Authors should describe the extent to which the investigators had access to the database population used to create the study population.<br><br>RECORD 12.2: Authors should provide information on the data cleaning methods used in the study.	Study design and databases<br><br>Figure 1
Linkage		..		RECORD 12.3: State whether the study included person-level, institutional-level, or other data linkage across two or more databases. The methods of linkage and methods of linkage quality evaluation should be provided.	Databases
**Results**					
Participants	13	(a) Report the numbers of individuals at each stage of the study (*e.g.*, numbers potentially		RECORD 13.1: Describe in detail the selection of the persons included in the study (*i.e.,* study population selection)	Figure 1 and Cohort Build

		eligible, examined for eligibility, confirmed eligible, included in the study, completing follow-up, and analysed)<br><br>(b) Give reasons for non-participation at each stage.<br><br>(c) Consider use of a flow diagram		including filtering based on data quality, data availability and linkage. The selection of included persons can be described in the text and/or by means of the study flow diagram.	
Descriptive data	14	(a) Give characteristics of study participants (*e.g.*, demographic, clinical, social) and information on exposures and potential confounders<br><br>(b) Indicate the number of participants with missing data for each variable of interest<br><br>(c) *Cohort study* - summarise follow-up time (*e.g.*, average and total amount)			Patient characteristics, Table 1, and Supplement
Outcome data	15	*Cohort study* - Report numbers of outcome events or summary measures over time<br><br>*Case-control study* - Report numbers in each exposure category, or summary measures of			Outcomes and Table 2

		exposure<br>*Cross-sectional study* - Report numbers of outcome events or summary measures			
Main results	16	(a) Give unadjusted estimates and, if applicable, confounder-adjusted estimates and their precision (e.g., 95% confidence interval). Make clear which confounders were adjusted for and why they were included<br>(b) Report category boundaries when continuous variables were categorized<br>(c) If relevant, consider translating estimates of relative risk into absolute risk for a meaningful time period			Tables 2-3
Other analyses	17	Report other analyses done—e.g., analyses of subgroups and interactions, and sensitivity analyses			Table 3 and Supplement
**Discussion**					
Key results	18	Summarise key results with			Discussion (start)

		reference to study objectives			
Limitations	19	Discuss limitations of the study, taking into account sources of potential bias or imprecision. Discuss both direction and magnitude of any potential bias		RECORD 19.1: Discuss the implications of using data that were not created or collected to answer the specific research question(s). Include discussion of misclassification bias, unmeasured confounding, missing data, and changing eligibility over time, as they pertain to the study being reported.	Discussion (end)
Interpretation	20	Give a cautious overall interpretation of results considering objectives, limitations, multiplicity of analyses, results from similar studies, and other relevant evidence			Discussion (end)
Generalisability	21	Discuss the generalisability (external validity) of the study results			Discussion (end)
**Other Information**					
Funding	22	Give the source of funding and the role of the funders for the present study and, if applicable, for the original study on which the present article is based			Disclosures
Accessibility of protocol, raw data,		..		RECORD 22.1: Authors should provide information on how to access any	Data access

and programming code				supplemental information such as the study protocol, raw data, or programming code.	statement

*Reference: Benchimol EI, Smeeth L, Guttmann A, Harron K, Moher D, Petersen I, Sørensen HT, von Elm E, Langan SM, the RECORD Working Committee. The REporting of studies Conducted using Observational Routinely-collected health Data (RECORD) Statement. *PLoS Medicine* 2015; in press.

*Checklist is protected under Creative Commons Attribution (CC BY) license.

Table S2: Data sources and codes used to define study criteria, exposures, outcomes, and covariates

Study Concept	Data Source/Code Type
Inclusion criteria	
Incident dialysis	Canadian Organ Replacement Register (CORR)
First dialysis session	Ontario Health Insurance Plan (OHIP)<br>Fee code: G082, G083, G085, G090-G096, G294, G295, G323, G325, G326, G330, G331, G333, G860-G866, H540, H740, R849<br>CCP: 5195, 6698<br>CCI: 1PZ21
Exclusion criteria	
Residence in a long-term care facility	Ontario Drug Benefit Claims
Kidney transplant in five years prior to index date	CORR
<3 primary care visits in two years prior to dialysis initiation	ICES Physician Database (IPDB)
Exposure	
Primary care physician	IPDB
Outcomes	
Time to Transplant	CORR
Time to Home Dialysis	CORR
Physician Visits	
GP/FP	IPDB; Main Specialty: "GP/FP"
Cardiology	IPDB; Main Specialty: "Cardiology"
Endocrinology	IPDB; Main Specialty: "Endocrinology"
Psychiatry	IPDB; Main Specialty: "Psychiatry"
Palliative Care	OHIP<br>Fee Code: A945, K023, G512, G511, B966, B998, B997, K700, C945, C882, C982, E083, K023, W872, W972, W882<br><br>Canadian Institute for Health Information Discharge Abstract Database (CIHI-DAD)<br>ICD-10: Z51.5

Study Concept	Data Source/Code Type
	National Ambulatory Care Reporting System (NACRS):<br>PRVSERV(1-10)=00121, CONSULTSERV1 to CONSULTSERV3 = 00121<br><br>Resident Assessment Instrument – Contact Assessment (RAI-CA):<br>B2c=1, B4=12<br><br>Resident Assessment Instrument – Home Care (RAI-HC):<br>P2S=1 or 2, CC3f goals of care=palliative care<br><br>Home Care Data (HCD):<br>SRC_admission=95, Service_RPC=95, Residence_type=2000, SRC_discharge=95<br><br>Continuing Care Reporting System (CCRS):<br>CCRS_P1A0=1
Cancer Screening Tests	
Mammogram (women only)	OHIP Fee Code: X185, X178<br>Ontario Breast Screening Program (OBSP)
PAP (women only)	OHIP Fee Code: G365, G394, E431, E430, L713, L733, L812, Q678
PSA (men only)	OHIP Fee Code: L354, L358
Colon Cancer	Colonoscopy: OHIP Fee Code Z555, Z491-Z499<br>FOBT: OHIP Fee Code L179A, L181A<br>Sigmoidoscopy: OHIP Fee Code Z580
Influenza Immunization	OHIP Fee Code: G590, G591, Q130
Medical Services Used	
Diabetes Assessment (DM only)	OHIP Fee Code: K030, Q040, K045, K046
Vision Test (DM only)	OHIP Fee Code: A110-A112, A114, A115, A233-A240, K065, K066, V401, V402, V404-V409, V450, V451
Covariates—Demographics	
Age	RPDB
Sex	RPDB
Ethnicity	CORR

Study Concept	Data Source/Code Type
Income quintile	RPDB
Rural residence	RPDB
Rostered to a primary care physician	Client Agency Program Enrolment database (CAPE)
Primary cause of kidney failure	CORR
Dialysis modality	CORR
Covariates—Healthcare utilization	
Physician visits	IPDB
Number of hospital days	CIHI-DAD
Home care	Home Care Database
Covariates—Comorbidities	
Diabetes (requires one inpatient or two outpatient claims)	CIHI-DAD<br>ICD-9: 250<br>ICD-10: E10, E11, E13, E14<br><br>OHIP<br>Diagnosis code: 250<br>Fee code: K029, K030, K045, K046, Q040
Heart failure (requires one inpatient or two outpatient claims)	CIHI-DAD<br>ICD-9: 425, 428, 514, 5184<br>ICD-10: I099, I255, I420, I425-I429, I43, I50, J81<br>CCP: 4961-4964<br>CCI: IHP53, IHP55, IHZ53GRFR, IHZ53LAFR, IHZ53SYFR<br><br>OHIP<br>Diagnosis code: 428<br>Fee code: R701, R702, Z429
Myocardial infarction	CIHI-DAD<br>ICD-9: 410<br>ICD-10: I21, I22
Stroke/transient ischemic attack	CIHI-DAD<br>ICD-9: 3623, 430, 431, 432, 434, 435, 436<br>ICD-10: G450-G453, G458, G459, H340, H341, I600-I607, I609, I61, I62, I630-I635, I638,

Study Concept	Data Source/Code Type
	I639, I64
Chronic obstructive pulmonary disease	CIHI-DAD<br>ICD-9: 491, 492, 496<br>ICD-10: J41, J43, J44
Hypertension	Ontario Hypertension database
Cancer	CIHI-DAD<br>ICD-9: V10, 140-149, 150-159, 160-165, 170-176, 179, 180-189, 190-194, 196-198, 1950-1955, 1958, 1990, 1991, 2000, 2001, 2002, 2008, 2010, 2011, 2012, 2014, 2015, 2016, 2017, 2019, 2020, 2026, 2028, 2029, 203-208, 230-234<br>ICD-10: C00-C09, C10-C19, C20-C26, C30-C34, C37-C39, C40-C41, C43-C49, C50-C58, C60-C69, C70-C79, C80-86, C8800, C8808, C90-C97, D00-D07, D09, Z850-Z859<br>80003, 80006, 80013, 80023, 80033, 80043, 80102, 80103, 80106, 80113, 80123, 80203, 80213, 80223, 80303, 80313, 80323, 80333, 80343, 80413, 80423, 80433, 80443, 80453, 80502, 80503, 80513, 80523, 80702, 80703, 80706, 80713, 80723, 80733, 80743, 80753, 80762, 80763, 80772, 80802, 80812, 80823, 80903, 80913, 80923, 80933, 80943, 80953, 81103, 81202, 81203, 81213, 81223, 81233, 81243, 81303, 81402, 81403, 81406, 81413, 81423, 81433, 81443, 81453, 81473, 81503, 81513, 81523, 81533, 81543, 81553, 81603, 81613, 81623, 81703, 81713, 81803, 81903, 82003, 82013, 82102, 82103, 82113, 82203, 82213, 82303, 82313, 82403, 82413, 82433, 82443, 82453, 82463, 82473, 82503, 82513, 82603, 82612, 82613, 82623, 82632, 82633, 82703, 82803, 82813, 82903, 83003, 83103, 83123, 83143, 83153, 83203, 83223, 83233, 83303, 83313, 83323, 83403, 83503, 83703, 83803, 83813, 83903, 84003, 84013, 84103, 84203, 84303, 84403, 84413, 84423, 84503, 84513, 84603, 84613, 84623, 84703, 84713, 84723, 84733, 84803, 84806, 84813, 84903, 84906, 85002, 85003, 85012, 85013, 85023, 85032, 85033, 85042, 85043, 85103, 85113, 85123, 85202, 85203, 85213, 85222, 85223, 85303, 85403, 85413, 85423, 85433, 85503, 85603, 85623, 85703, 85713, 85723, 85733, 85803, 86003, 86203, 86303, 86403, 86503, 86803, 86933, 87003, 87103, 87202, 87203, 87213, 87223, 87233, 87303, 87403, 87412, 87413, 87422, 87423, 87433, 87443, 87453, 87613, 87703, 87713, 87723, 87733, 87743, 87803, 88003, 88006, 88013, 88023, 88033, 88043, 88103, 88113, 88123, 88133, 88143, 88303, 88323, 88333, 88403, 88503, 88513, 88523, 88533, 88543, 88553, 88583, 88903, 88913, 88943, 88953, 88963, 89003, 89013, 89023, 89103, 89203, 89303, 89333, 89403, 89413, 89503, 89513, 89603, 89633, 89643, 89703, 89713, 89723, 89803, 89813, 89903, 89913, 90003, 90203, 90403, 90413, 90423, 90433, 90443, 90503, 90513, 90523, 90533, 90603, 90613, 90623, 90633, 90643, 90703, 90713, 90723,

Study Concept	Data Source/Code Type
	90803, 90813, 90823, 90833, 90843, 90853, 90903, 91003, 91013, 91023, 91103, 91203, 91243, 91303, 91333, 91403, 91503, 91703, 91803, 91813, 91823, 91833, 91843, 91853, 91903, 92203, 92213, 92303, 92313, 92403, 92503, 92513, 92603, 92613, 92703, 92903, 93103, 93303, 93623, 93643, 93703, 93803, 93813, 93823, 93903, 93913, 93923, 94003, 94013, 94103, 94113, 94203, 94213, 94223, 94233, 94243, 94303, 94403, 94413, 94423, 94433, 94503, 94513, 94603, 94703, 94713, 94723, 94733, 94803, 94813, 94903, 95003, 95013, 95023, 95033, 95043, 95103, 95113, 95123, 95203, 95213, 95223, 95233, 95303, 95393, 95403, 95603, 95613, 95803, 95813, 95903, 95913, 95933, 95943, 95953, 96503, 96523, 96533, 96543, 96553, 96573, 96583, 96593, 96603, 96613, 96623, 96633, 96643, 96653, 96663, 96673, 96703, 96713, 96723, 96733, 96743, 96753, 96763, 96773, 96803, 96813, 96823, 96833, 96843, 96853, 96863, 96873, 96903, 96913, 96923, 96933, 96943, 96953, 96963, 96973, 96983, 97003, 97013, 97023, 97053, 97063, 97073, 97093, 97113, 97123, 97133, 97143, 97203, 97223, 97233, 97313, 97323, 97403, 97413, 97603, 97613, 97623, 97633, 97643, 98003, 98013, 98023, 98033, 98043, 98203, 98213, 98223, 98233, 98243, 98253, 98263, 98273, 98303, 98403, 98413, 98423, 98503, 98603, 98613, 98623, 98633, 98643, 98663, 98673, 98683, 98703, 98803, 98903, 98913, 98923, 98933, 98943, 99003, 99103, 99303, 99313, 99323, 99403, 99413<br><br>OHIP<br>Diagnosis code: 140-149, 150-159, 160-165, 170-175, 179, 180-189, 190-199, 200-208, 230-234
Chronic liver disease	CIHI-DAD<br>ICD-9: 070, 2750, 2751, 4561, 4562, 571, 5722, 5723, 5724, 5728, 573, 7824, 7891, 7895, V026<br>ICD-10: B16-19, B942, E831, E830, I85, K70, K713, K714, K715, K717, K721, K729, K73, K74, K753, K754, K758, K759, K76, K77, R160, R162, R17, R18, Z225<br><br>OHIP<br>Diagnosis code: 070, 571, 573<br>Fee code: Z551, Z554
Peripheral vascular disease	CIHI-DAD<br>ICD-9: 4439, 4402, 4408, 4409, 444, 5571<br>ICD-10: I700, I702, I708, I709, I731, I738, I739, K551<br>CCP: 5014, 5016, 5018, 5028, 5038, 5125, 5126, 5129, 5159<br>CCI: 1KA50, 1KA76, 1KE76, 1KG50, 1KG57, 1KG76MI, 1KG87, 1IA87LA, 1IB87LA,

Study Concept	Data Source/Code Type
	1IC87LA, 1ID87LA, 1KA87LA, 1KE57<br><br>OHIP<br>Fee code: E626, E649, E672, R780, R783-R787, R791, R794, R797, R804, R809, R813-R815, R855, R856, R860, R861, R867, R875, R933, R934, R936, R937
Depression (Defined as 1 hospitalization or claim in 2 years or less)	CIHI-DAD/National Ambulatory Care Reporting System (NACRS)<br>ICD-9: 2962, 2963, 2965, 3004, 309, 311<br>ICD-10: F204, F313-F315, F32, F33, F341, F412, F432
Chronic pain (Defined as 2 hospitalizations or 2 claims or 2 NACRS claims in 30 days or less)	CIHI-DAD/NACRS<br>ICD-9: 7200, 7202, 7209, 7210-7214, 7216, 7218, 7219, 722, 7230, 7231, 7233-7239, 7240-7246, 7248, 7249, 7290, 7291, 7292, 7294, 7295<br>ICD-10: F454, M081, M2550, M2551, M2555, M2556, M2557, M432-M436, M45, M461, M463, M464, M469, M47, M480, M481, M488, M489, M508, M509, M51, M531, M532, M533, M538, M539, M54, M608, M609, M633, M790, M791, M792, M796, M797, M961
Dementia (Defined as 1 hospitalization, or 2 claims in 2 years or less)	CIHI-DAD<br>ICD-9: 2900-2904, 2908, 2909, 2941, 2948, 2949, 3310, 3311, 3312, 797<br>ICD-10: F00-F03, F09, F051, F065, F066, F068, F069, G30, G31, R54<br><br>OHIP<br>Diagnosis code: 290, 331, 797

Table S3: Characteristics of patients initiating maintenance hemodialysis in Ontario, Canada, propensity matched on the likelihood of high primary care physician continuity

Baseline Characteristics	Low Continuity (n=9530)	High Continuity (n=9530)	Standardized Difference
Demographics			
Age (years), median (IQR)	67 (56-77)	67 (56-77)	0.01
Female, n (%)	3666 (38.5)	3666 (38.5)	0.00
Ethnicity, n (%)			
Caucasian	6688 (70.2)	6728 (70.6)	0.01
Indian subcontinent	673 (7.1)	663 (7.0)	0.004
Black	525 (5.5)	519 (5.5)	0.003
Asian	500 (5.3)	493 (5.2)	0.003
Other/Unknown	1144 (12.0)	1127 (11.8)	0.01
Income quintile, n (%)			
1 (lowest income)	2424 (25.4)	2479 (26.0)	0.01
2	2149 (22.6)	2086 (21.9)	0.02
3	1818 (19.1)	1872 (19.6)	0.01
4	1635 (17.2)	1654 (17.4)	0.01
5 (highest income)	1461 (15.3)	1402 (14.7)	0.02
Rural residence, n (%)	1115 (11.7)	1138 (11.9)	0.01
Rostered to a primary care physician, n (%)	8009 (84.0)	8009 (84.0)	0.00
Primary cause of kidney failure, n (%)			
Diabetes	3489 (36.6)	3454 (36.2)	0.01
Renal vascular disease	1439 (15.1)	1468 (15.4)	0.01
Glomerulonephritis/autoimmune	1039 (10.9)	1040 (10.9)	0.00
Cystic kidney disease	305 (3.2)	323 (3.4)	0.01
Other	1972 (20.7)	1988 (20.9)	0.004
Unknown	1286 (13.5)	1257 (13.2)	0.01
Year of cohort entry, n (%)			
2006-2007	630 (6.6)	649 (6.8)	0.008
2008	602 (6.3)	617 (6.5)	0.01
2009	624 (6.6)	689 (7.2)	0.03

**Baseline Characteristics**	**Low Continuity (n=9530)**	**High Continuity (n=9530)**	**Standardized Difference**
2010	715 (7.5)	730 (7.7)	0.01
2011	779 (8.2)	763 (8.0)	0.01
2012	850 (8.9)	805 (8.5)	0.02
2013	957 (10.0)	898 (9.4)	0.02
2014	1057 (11.1)	992 (10.4)	0.02
2015	1125 (11.8)	1099 (11.5)	0.01
2016	1178 (12.4)	1081 (11.3)	0.03
2017	1013 (10.6)	1207 (12.7)	0.06
Comorbidities, n (%)			
Cancer	3571 (37.5)	3586 (37.6)	0.003
Chronic liver disease	1056 (11.1)	1036 (10.9)	0.01
Chronic obstructive pulmonary disease	1064 (11.2)	1071 (11.2)	0.002
Chronic pain	275 (2.9)	275 (2.9)	0.000
Congestive heart failure	3349 (35.1)	3396 (35.6)	0.01
Dementia	451 (4.7)	449 (4.7)	0.001
Depression	314 (3.3)	325 (3.4)	0.01
Diabetes	5604 (58.8)	5604 (58.8)	0.000
Hypertension	8573 (90.0)	8613 (90.4)	0.01
Myocardial infarction	992 (10.4)	995 (10.4)	0.001
Peripheral vascular disease	670 (7.0)	658 (6.9)	0.01
Stroke/transient ischemic attack	646 (6.8)	632 (6.6)	0.01
Modified Charlson comorbidity index, mean (SD)	3.8 (1.9)	3.8 (1.9)	0.01
Healthcare utilization in previous year, median (IQR)			
No. of visits to all primary care physicians	8 (4-15)	9 (5-15)	0.04
No. of visits to most frequent primary care physician	4 (2-8)	8 (4-13)	0.63
No. of internal medicine visits	2 (0-6)	2 (0-6)	0.04
No. of nephrology visits	6 (3-10)	6 (3-10)	0.01
No. of endocrinology visits	0 (0-0)	0 (0-0)	0.02
No. of cardiology visits	2 (1-5)	2 (1-5)	0.04
No. of geriatric medicine visits	0 (0-0)	0 (0-0)	0.01

Baseline Characteristics	Low Continuity (n=9530)	High Continuity (n=9530)	Standardized Difference
No. of psychiatry visits	0 (0-0)	0 (0-0)	0.02
No. of hospitalization days over prior year	1 (0-10)	1 (0-9)	0.07
Home care, n (%)	2782 (29.2)	2831 (29.7)	0.01
Physician characteristics			
Age (years), mean (SD)	53.6 (11.6)	53.7 (10.3)	0.01
Male, n (%)	6991 (73.4)	7017 (73.6)	0.01
Number of years since graduation, mean (SD)	27.4 (12.2)	27.5 (10.8)	0.01
International medical graduate, n (%)	2314 (24.3)	2291 (24.0)	0.01
Hospital affiliation, n (%)	3540 (37.2)	3558 (37.3)	0.004
Rural practice, n (%)	893 (9.4)	939 (9.9)	0.02
Dialysis patient volume in previous year, n (%)			
0	1680 (17.6)	1682 (17.7)	0.001
1	1977 (20.8)	1965 (20.6)	0.003
2	1679 (17.6)	1689 (17.7)	0.003
3	1260 (13.2)	1296 (13.6)	0.01
4	853 (9.0)	826 (8.7)	0.01
≥5	2081 (21.8)	2072 (21.7)	0.002

Healthcare utilization in previous year with 0 median visits, summarized as categorical variables, n (%)
No. of endocrinology visits: Low continuity 0=7166 (75.2), 1=743 (7.8), 2=579 (6.1), ≥3=1042 (10.9); High continuity 0=7228 (75.8), 1=722 (7.6), 2=604 (6.3), ≥3=976 (10.2)
No. of geriatric medicine visits: Low continuity 0=8817 (92.5), 1=303 (3.2), 2=137 (1.4), ≥3=273 (2.9); High continuity 0=8830 (92.7), 1=307 (3.2), 2=124 (1.3), ≥3=269 (2.8)
No. of psychiatry visits: Low continuity 0=8923 (93.6), 1=221 (2.3), 2=100 (1.1), ≥3=286 (3.0); High continuity 0=8968 (94.1), 1=205 (2.2), 2=92 (1.0), ≥3=265 (2.8)

Table S4: Medical service utilization among patients surviving on maintenance hemodialysis for 5 years duration

Medical Services	Patients Surviving 5-years on Maintenance Dialysis (n=4014)	
	Event rate per patient-year (95% CI)	No. (%) with 0 events
**Specialist Visits**		
Nephrology	50.70 (50.62-50.79)	<6 (<1)
Cardiology	3.34 (3.32-3.37)	65 (1.6)
Endocrinology	0.64 (0.63-0.65)	2117 (52.7)
Psychiatry	0.43 (0.43-0.44)	3000 (74.7)
Palliative care	1.58 (1.56-1.59)	2759 (68.7)
**Cancer Screening**		
Mammography (women only)	0.16 (0.16-0.17)	768 (46.9)
Papanicolaou testing (women only)	0.16 (0.15-0.17)	983 (60.1)
Prostate-specific antigen testing (men only)	0.07 (0.06-0.07)	1936 (81.5)
Colon cancer	0.09 (0.09-0.09)	2415 (60.2)
**Other Preventative Care**		
Influenza immunization	0.25 (0.24-0.25)	1828 (45.5)
Diabetes assessment (patients with diabetes only)	0.47 (0.46-0.48)	1071 (48.2)
Diabetes vision screening (patients with diabetes only)	1.36 (1.34-1.38)	575 (25.9)
